# Microscopic nonlinear optical response: Analysis and calculations with the Floquet–Bloch formalism

**DOI:** 10.1063/4.0000220

**Published:** 2024-02-23

**Authors:** Daria Popova-Gorelova, Robin Santra

**Affiliations:** 1 Center for Free-Electron Laser Science CFEL, Deutsches Elektronen-Synchrotron DESY, Notkestr. 85, 22607 Hamburg, Germany; 2 Department of Physics, Universität Hamburg, Notkestr. 9, D-22607 Hamburg, Germany; 3 The Hamburg Centre for Ultrafast Imaging, Universität Hamburg, Luruper Chaussee 149, D-22761 Hamburg, Germany

## Abstract

We analyze microscopic nonlinear optical response of periodic structures within the Floquet–Bloch formalism. The analysis is focused on the real-space distributions of optically induced charge and electron current density within the unit cell of a crystal. We demonstrate that the time-reversal symmetry of a crystal determines the phases of the temporal oscillations of these distributions. We further analyze their spatial symmetries and connection to macroscopic optical response. We illustrate our study with *ab initio* calculations that combine density functional theory with the Floquet–Bloch formalism. The calculations provide time-dependent optically induced charge distributions and electron current densities within the unit cells of a crystal with inversion symmetry MgO and a crystal without inversion symmetry GaAs in response to a strong-field excitation. The real-space, microscopic view on nonlinear optical response provides insightful information about the strong field–matter interaction.

## I. INTRODUCTION

Strong-field excitation by light can be used to induce various important mechanisms in solids, such as manipulation of electronic gaps and structure by light,^1–12^ or generation of high harmonics (HHG).^13^ Such processes, on the one hand, have a big potential for the development of petahertz electronics and, on the other hand, raise many scientific questions about the mechanisms behind them.^14,15^ Access to microscopic properties of laser-driven electron dynamics is necessary for a deeper understanding of strong-field phenomena in solids.^14,16–22^

The optical response of crystals has been extensively investigated for more than a hundred years. Such studies have predominantly concentrated on the macroscopic optical response of a crystal, since it determines typical experimentally detectable observables, such as harmonic generation. The macroscopic optical response of a crystal in most cases results from an induced dipole moment. Typically, the radiation power produced by the oscillating dipole moment dominates over the radiation power produced by higher-order moments in the optical regime,^23^ and the macroscopic polarization in a dielectric material results from the induced dipole moment.^24^ For this reason, it is customary to relate linear and nonlinear optical response to the induction of dipole moments.

In recent years, laser-driven electron dynamics has gained considerable attention due to remarkable achievements in the field of nonlinear optics including the generation of high harmonics (HHG) in solids,^13,25^ optical-field-induced currents in dielectrics,^1^ manipulation of electric properties of a dielectric with the electric field of light,^2^ control of coherent Bloch oscillations,^5,6^ subcycle terahertz nonlinear optical effects,^3^ and coherent control of currents in semiconductors using synthesized optical waveforms.^4^ These achievements motivated theoretical and experimental studies to understand the mechanisms behind these phenomena.^12,15–17,26–38^

We employ the Floquet–Bloch framework, which provides us with a material-specific description of light–matter interaction beyond perturbation theory,^39–41^ and is also a convenient tool to analyze properties of laser-dressed systems, such as selection rules of HHG in periodic structures.^30,39,42,43^ Here, employing the Floquet–Bloch formalism, we analyze spatial symmetry and temporal behavior of microscopic optically induced charge and electron-current distributions.

The Floquet formalism implies that the electric field of the driving field is temporally periodic. It has been shown in Refs. 44 and 45 that this approximation is already justified for a strong-field optical field with a duration comprising several tens of optical cycles. Throughout this paper, we consider a laser-dressed crystal in a state that is characterized by a single Floquet state. To justify this approximation, it should be assured that the driving field is not too strong to bring the laser-dressed system into a superposition of several Floquet states.^46^ The applicability of these approximations can be verified with the radiation spectrum generated by the system through the driving field. If they do not hold, the radiation spectrum will have additional peaks besides the harmonic ones. Thus, our study applies to the regime in which each generated radiation peak can be clearly assigned to an integer multiple of the driving-laser frequency.

The article is organized as follows. We analyze in Sec. II temporal and spatial properties of the microscopic nonlinear optical response within the Floquet–Bloch formalism. Namely, we focus on analysis of optically induced charge distribution and electron current density in real space. In Sec. III, we provide details of our calculation scheme based on the density functional theory (DFT) combined with the Floquet–Bloch formalism to calculate microscopic nonlinear optical response. We calculate the microscopic optical response of two bandgap materials, the insulator MgO, and the semiconductor GaAs driven by an intense optical field. By studying these two prototypical materials, we cover two types of crystals, one without inversion symmetry, GaAs, and one with inversion symmetry, MgO.

## II. MICROSCOPIC OPTICALLY INDUCED CHARGE AND ELECTRON-CURRENT DISTRIBUTIONS

We briefly review the classical limit of the quantized Floquet–Bloch formalism that we use to describe the nonperturbative interaction of an optical electromagnetic field with a bandgap crystal.^39–41,47–49^ The Hamiltonian of a laser-dressed crystal is given by

$${\hat{H}}_{\text{el}-\text{em}}={\hat{H}}_{\text{el}}+{\hat{H}}_{\text{int}}+{\hat{H}}_{\text{em}},$$ (1)

$${\hat{H}}_{\text{el}}=\int d^{3}r{\hat{\psi }}^{†}(r)[p^{2}/2+V_{c}(r)]\hat{\psi }(r),$$ (2)

$${\hat{H}}_{\text{em}}=\omega {\hat{a}}_{\kappa_{0},s_{0}}^{†}{\hat{a}}_{\kappa_{0},s_{0}},$$ (3)

$${\hat{H}}_{\text{int}}=\bar{\alpha }\int d^{3}r{\hat{\psi }}^{†}(r)\left({\hat{A}}_{\text{em}}(r)\cdot p\right)\hat{\psi }(r).$$ (4)

Here, ${\hat{H}}_{\text{el}}$ is the mean-field Hamiltonian of the unperturbed crystal with one-body eigenstates $\left|\varphi_{mk\sigma }\right\rangle$, where **k** is the Bloch wave vector, *m* is the band, and *σ* is the spin index. $V_{c}(r)=V_{c}(r+R)$ is a space-periodic crystal field potential, and R is a lattice vector. According to the Bloch theorem,^50^ the corresponding one-body wave function of $\left|\varphi_{mk\sigma }\right\rangle$ has the form $\varphi_{mk\sigma }(r)=e^{ik\cdot r}u_{mk\sigma }(r)$, where $u_{mk\sigma }(r)=u_{mk\sigma }(r+R)$ is a space-periodic function. p is the canonical momentum of an electron, and ${\hat{\psi }}^{†}$ ( $\hat{\psi }$) is the electron creation (annihilation) field operator. ${\hat{H}}_{\text{em}}$ is the Hamiltonian of the electromagnetic field, and ${\hat{H}}_{\text{int}}$ the interaction Hamiltonian between the electromagnetic field and the electronic system, which we describe within the dipole approximation. ${\hat{a}}_{\kappa ,s}^{†}$ ( ${\hat{a}}_{\kappa ,s}$) creates (annihilates) a photon with wave vector κ and polarization *s*. We assume that only the $\kappa_{0}$, *s*_0_ mode with a corresponding polarization vector $\epsilon_{0}$ and the energy $\omega =|\kappa_{0}|c$, where *c* is the speed of light, is occupied in the driving electromagnetic field, and that the state of the field is described by a single-mode coherent state |α,t⟩. ${\hat{A}}_{\text{em}}(r)$ is the vector potential operator of the electromagnetic field, and $\bar{\alpha }$ is the fine-structure constant. We use atomic units for these and the following expressions.

Since the state of the electromagnetic field |α,t⟩ is unaffected by the interaction with the electronic system by assumption, the one-body solution of the time-dependent Schrödinger equation $id|\psi_{i,k\sigma },t\rangle /dt={\hat{H}}_{\text{el}-\text{em}}|\psi_{i,k\sigma },t\rangle$ can be represented as $\left|\psi_{i,k\sigma },t\rangle =|\phi_{i,k\sigma }^{\text{el}},t\rangle |\alpha ,t\right\rangle$ with the corresponding electronic one-body Floquet–Bloch wave function^39,40,48^

$$\phi_{i,k\sigma }^{\text{el}}(r,t)=\sum_{m,\mu }c_{m,k\sigma ,\mu }^{i}e^{-i\mu \omega t}\varphi_{mk\sigma }(r),$$ (5)

where *μ* is an integer and the $c_{m,k\sigma ,\mu }^{i}$ are expansion coefficients.

### A. Optically induced charge distributions

Within the Floquet formalism, the electron density of the laser-dressed system evolves in time as^49^

$$\rho (r,t)=\sum_{\mu }e^{i\mu \omega t}{\tilde{\rho }}_{\mu }(r),$$ (6)

with *μ*th-order optically induced charge distributions

$${\tilde{\rho }}_{\mu }(r)=\sum_{\sigma }\int_{\text{BZ}}\frac{d^{3}k}{V_{\text{uc}}}\sum_{m,m\prime ,i,\mu \prime }c_{m^{\prime },k\sigma ,\mu^{\prime }+\mu }^{i*}c_{m,k\sigma ,\mu^{\prime }}^{i}u_{m^{\prime }k\sigma }^{†}(r)u_{mk\sigma }(r),$$ (7)

where $V_{\text{uc}}$ is the volume of a unit cell, *i* denotes the index of occupied one-body Floquet–Bloch states, and the integration is over the Brillouin zone. The *μ*th-order optically induced charge distributions can be connected to properties of the *μ*th-order macroscopic optical response. For example, the polarization is determined by the dipole moment of the time-dependent electron density

$$P(t)\propto \int d^{3}rr\rho (r,t),$$ (8)

and the *μ*th-order component of the polarization is determined by the *μ*th-order optically induced charge distribution

$${\tilde{P}}^{\left(\mu \right)}(\mu \omega )\propto e^{i\mu \omega t}\int d^{3}rr{\tilde{\rho }}_{\mu }(r).$$ (9)

Thus, the amplitudes ${\tilde{\rho }}_{\mu }(r)$ are optically induced charge distributions that give rise to a *μ*th-order macroscopic optical response.

Our connection of the optically induced charge distributions derived within the Floquet–Bloch formalism to the macroscopic polarization is consistent with the perturbative derivation of macroscopic polarization by Bloembergen and Shen.^51,52^ Their expansion of the polarization in orders of *ω*, $P={\tilde{P}}^{\left(1\right)}(\omega )+{\tilde{P}}^{\left(2\right)}(2\omega )+{\tilde{P}}^{\left(3\right)}(3\omega )+\cdots$ also holds within the Floquet formalism. However, *μ*th-order components derived within the Floquet formalism do not have to scale as the *μ*th power of the electric-field amplitude, in contrast to the ones derived within the perturbation theory by Bloembergen and Shen.

The volume integral of a *μ*th-order optically induced charge distribution over a unit cell is given by

$$\int d^{3}r{\tilde{\rho }}_{\mu }(r)=\sum_{\sigma }\int_{\text{BZ}}\frac{d^{3}k}{V_{\text{uc}}}\sum_{m,m\prime ,i,\mu \prime }c_{m^{\prime },k\sigma ,\mu^{\prime }+\mu }^{i*}c_{m,k\sigma ,\mu^{\prime }}^{i}\delta_{m,m\prime },$$ (10)

where the sum goes over spin directions *σ*. We now apply the orthogonality of the expansion coefficients $\sum_{m,\mu \prime }c_{m,k\sigma ,\mu^{\prime }+\mu }^{i*}c_{m,k\sigma ,\mu^{\prime }}^{i}=\sum_{m,\mu \prime }\delta_{\mu \prime +\mu ,\mu \prime }$ and obtain

$$\int d^{3}r{\tilde{\rho }}_{\mu }(r)=N_{\text{el}}\delta_{\mu ,0}.$$ (11)

The volume integral of the optically induced charge distributions of a nonzero order that enter in the time-dependent part of ρ(r,t) is zero, $\int d^{3}r{\tilde{\rho }}_{\mu \neq 0}(r)=0$. These relations indicate that the interaction of a crystal with light leads to a dynamical redistribution of charges, which has positive and negative regions relative to the field-free electron density. These positive and negative regions coherently oscillate and cancel each other when volume integrated. The positively charged regions are due to electron holes in valence bands, and the negatively charged regions are due to electrons in conduction bands.

### B. Time-reversal symmetry and Floquet–Bloch wave functions

In the following, we will show that the time-reversal symmetry of a crystal determines the temporal behavior of the laser-driven spin-averaged electron oscillations. The derivations below are applied to a general situation in which the crystal is not necessarily invariant under inversion symmetry in real space. First, let us consider how time-reversal symmetry influences the properties of the Floquet–Bloch functions. For a Bloch function $\varphi_{mk↑}(r)$ of an unperturbed crystal that obeys time-reversal symmetry, it is valid that $\varphi_{mk↑}(r)=\varphi_{m-k↓}^{*}(r)$.^50^ Similar to the proof of this property, we show below that a one-body electronic wave function of a laser-dressed crystal with time-reversal symmetry obeys

$$\phi_{i,k↑}^{\text{el}}(r,t)=\phi_{i,-k↓}^{*\text{el}}(r,T/2-t),$$ (12)

where T=2π/ω is the period of the driving electromagnetic field.

Applying to the time-dependent Schrödinger equation

$$\begin{array}{c} i\frac{d|\phi_{i,k↑}^{\text{el}},t\rangle }{dt}|\alpha ,t\rangle +i|\phi_{i,k↑}^{\text{el}},t\rangle \frac{d|\alpha ,t\rangle }{dt}=({\hat{H}}_{\text{el}}+{\hat{H}}_{\text{int}}+{\hat{H}}_{\text{em}})|\phi_{i,k↑}^{\text{el}},t\rangle |\alpha ,t\rangle \end{array}$$ (13)

that the coherent state |α,t⟩ obeys $id|\alpha ,t\rangle /dt={\hat{H}}_{\text{em}}|\alpha ,t\rangle$, and multiplying Eq. (13) by ⟨α,t|, we obtain

$$i\frac{d|\phi_{i,k↑}^{\text{el}},t\rangle }{dt}={\hat{H}}_{\text{el}}|\phi_{i,k↑}^{\text{el}},t\rangle +\langle \alpha ,t|{\hat{H}}_{\text{int}}|\alpha ,t\rangle |\phi_{i,k↑}^{\text{el}},t\rangle .$$ (14)

The matrix element $\left\langle \alpha ,t|{\hat{H}}_{\text{int}}|\alpha ,t\right\rangle$ gives the interaction Hamiltonian in the classical limit ${\hat{H}}_{\text{int}}^{\text{cl}}(t)=\bar{\alpha }\int d^{3}r{\hat{\psi }}^{†}(r)\left(A_{\text{em}}(r_{0},t)\cdot p\right)\hat{\psi }(r)$, where $A_{\text{em}}(r_{0},t)=(c/\omega )E_{\text{em}}(r_{0})\;\text{cos}(\omega t)$ with $E_{\text{em}}(r_{0})$ being the amplitude of the electric field. Since we apply the dipole approximation, we ignored the spatial variation of the vector potential and the electric-field amplitude, and substituted the position of the crystal $r_{0}$ for **r** in $A_{\text{em}}(r_{0},t)$ and $E_{\text{em}}(r_{0})$. Thus, the one-body electronic wave function obeys

$$i\frac{\partial \phi_{i,k↑}^{\text{el}}(r,t)}{\partial t}=[{\hat{H}}_{\text{el}}+{\hat{H}}_{\text{int}}^{\text{cl}}(t)]\phi_{i,k↑}^{\text{el}}(r,t).$$ (15)

In order to prove Eq. (12), we take the complex conjugate of Eq. (15) and use that ${\hat{H}}_{\text{int}}^{\text{cl}*}(t)=-{\hat{H}}_{\text{int}}^{\text{cl}}(t)$resulting in

$$-i\frac{\partial \phi_{i,k↑}^{\text{el}*}(r,t)}{\partial t}=[{\hat{H}}_{\text{el}}^{*}-{\hat{H}}_{\text{int}}^{\text{cl}}(t)]\phi_{i,k↑}^{\text{el}*}(r,t).$$ (16)

We apply the operator ${\hat{\sigma }}_{s}=i{\hat{\sigma }}_{y}$, where ${\hat{\sigma }}_{y}$ is a Pauli matrix, which acts only on spin on both sides of the equation above

$$i\frac{\partial \phi_{i,k↓}^{\text{el}*}(r,t)}{\partial t}=[{\hat{\sigma }}_{s}{\hat{H}}_{\text{el}}^{*}-{\hat{\sigma }}_{s}{\hat{H}}_{\text{int}}^{\text{cl}}(t)]\phi_{i,k↑}^{\text{el}*}(r,t).$$ (17)

We further use that ${\hat{H}}_{\text{int}}^{\text{cl}}(t)$ commutes with ${\hat{\sigma }}_{s}$, and ${\hat{H}}_{\text{el}}^{*}=\sigma_{s}^{†}{\hat{H}}_{\text{el}}{\hat{\sigma }}_{s}$ for crystals with time-reversal symmetry,^50^ which results in

$$i\frac{\partial \phi_{i,k↓}^{\text{el}*}(r,t)}{\partial t}=[{\hat{H}}_{\text{el}}-{\hat{H}}_{\text{int}}^{\text{cl}}(t)][-\phi_{i,k↓}^{\text{el}*}(r,t)].$$ (18)

We now rewrite the above expression for the time T/2−t taking into account that $A_{\text{em}}(r_{0},T/2-t)=-A_{\text{em}}(r_{0},t)$ and that the derivative over time changes it sign for a negative time

$$i\frac{\partial \phi_{i,k↓}^{\text{el}*}(r,T/2-t)}{\partial t}=[{\hat{H}}_{\text{el}}+{\hat{H}}_{\text{int}}^{\text{cl}}(t)]\phi_{i,k↓}^{\text{el}*}(r,T/2-t).$$ (19)

Thus, we obtain that if the wave function $\phi_{i,k↑}^{\text{el}}(r,t)$ is a solution of the time-dependent Schrödinger equation, then $\phi_{i,k↓}^{\text{el}*}(r,T/2-t)$ is also a solution. Since $\phi_{i,k↑}^{\text{el}}(r+R,t)=e^{ik\cdot R}\phi_{i,k↑}^{\text{el}}(r,t)$, it must be valid that ${\hat{\sigma }}_{s}\phi_{i,k↑}^{\text{el}*}(r+R,T/2-t)=e^{-ik\cdot R}{\hat{\sigma }}_{s}\phi_{i,k↑}^{\text{el}*}(r,T/2-t)$. Thus, the solution $\phi_{i,k↓}^{\text{el}*}(r,T/2-t)$ is the solution at the Bloch vector −k, and we have proven Eq. (12). Equation (12) leads to a connection between the corresponding expansion coefficients of the Floquet–Bloch functions [cf. Eq. (5)]

$$c_{m,k↑,\mu }^{i}={\left(-1\right)}^{\mu }{\left(c_{m,-k↓,\mu }^{i}\right)}^{*},$$ (20)

which follows from the phase relation $e^{i\mu \omega t}={\left(-1\right)}^{\mu }e^{-i\mu \omega (T/2-t)}$. The relation in Eq. (20) is necessary for the analysis of the temporal dependence of optically induced charge distributions and electron current densities.

### C. Oscillations of optically induced charge distributions

We now make use of Eq. (20) and the property of the Bloch functions of crystals with time-reversal symmetry that $u_{mk↑}(r)=u_{m-k↓}^{*}(r)$^50^ to connect terms with opposite **k** in the integral for ${\tilde{\rho }}_{\mu }(r)$ in Eq. (7). This allows us to reduce the integration over the Brillouin zone to half of the Brillouin zone (HBZ) for the calculation of spin-averaged electron density in the absence of spin–orbit coupling (SOC), which lifts the degeneracy of the spin components of electronic states

$$\begin{array}{c} {\tilde{\rho }}_{\mu }(r)=\sum_{\sigma }\int_{\text{HBZ}}\frac{d^{3}k}{V_{\text{uc}}}\sum_{m,m\prime ,i,\mu \prime }c_{m^{\prime },k\sigma ,\mu^{\prime }+\mu }^{i*}c_{m,k\sigma ,\mu^{\prime }}^{i}u_{m^{\prime }k\sigma }^{†}(r)u_{mk\sigma }(r)+{\left(-1\right)}^{\mu }c.c. \end{array}$$ (21)

It follows from this relation that even-order optically induced spin-averaged charge distributions ${\tilde{\rho }}_{\mu_{\text{even}}}(r)$ are real functions, whereas odd-order optically induced spin-averaged charge distributions ${\tilde{\rho }}_{\mu_{\text{odd}}}(r)$ are purely imaginary. Spin-resolved charge distributions in the presence of spin–orbit coupling have both nonzero real and imaginary parts, which are connected via ${\tilde{\rho }}_{\mu_{\text{odd}}↑}(r)=-{\tilde{\rho }}_{\mu_{\text{odd}}↓}^{*}(r)$ and ${\tilde{\rho }}_{\mu_{\text{even}}↑}(r)={\tilde{\rho }}_{\mu_{\text{even}}↓}^{*}(r)$. However, upon spin averaging, the imaginary or real parts of even or odd amplitudes, respectively, vanish. In the following, electron densities and electron current densities are spin-averaged unless stated otherwise.

The time-reversal symmetry has an important consequence for the time dependence of the electron density. We use Eq. (21) to combine terms with opposite *μ* in the expression for the time-dependent electron density in Eq. (6). It can also be easily derived that ${\tilde{\rho }}_{\mu }(r)={\tilde{\rho }}_{-\mu }^{*}(r)$ independently of the crystal symmetry. This results in the time dependence of the electron density

$$\begin{array}{c} \rho (r,t)={\tilde{\rho }}_{0}(r)-\varrho_{1}(r)\;\text{sin}(\omega t)+\varrho_{2}(r)\;\text{cos}(2\omega t)-\varrho_{3}(r)\;\text{sin}(3\omega t)+\cdots \\ ={\tilde{\rho }}_{0}(r)-\sum_{\mu_{\text{odd}}\geq 1}\varrho_{\mu_{\text{odd}}}\;\text{sin}(\mu_{\text{odd}}\omega t)+\sum_{\mu_{\text{even}}\geq 2}\varrho_{\mu_{\text{even}}}\;\text{cos}(\mu_{\text{even}}\omega t), \end{array}$$ (22)

for the driving field with the vector potential evolving as cos(ωt). In Eq. (22), we redefined the optically induced charge distributions as follows:

$$\varrho_{\mu_{\text{even}}}(r)=2\text{Re}[{\tilde{\rho }}_{\mu_{\text{even}}}(r)],$$ (23)

$$\varrho_{\mu_{\text{odd}}}(r)=2\text{Im}[{\tilde{\rho }}_{\mu_{\text{odd}}}(r)],$$ (24)

which are real functions for both even and odd *μ*. This representation of optically induced charge distributions is more insightful in comparison with the functions ${\tilde{\rho }}_{\mu }(r)$, since it demonstrates the actual time dependence of the light-induced charge distributions. We, thus, obtain that time-reversal symmetry determines the phases of *μ*th-order oscillations of optically induced charge distributions. Broken time-reversal symmetry would lead to a different relation between the phases of induced-charge and electric-field oscillations.

### D. Oscillations of electron-current-density amplitudes

We now analyze time dependence of the electron current density. The electron current density in the presence of the electromagnetic field is given by^39,53^

$$j(r,t)=-\rho (r,t)A_{\text{em}}(r_{0},t)+\int_{\text{BZ}}\frac{d^{3}k}{V_{\text{uc}}}\text{Im}\left[\sum_{i,\sigma }\phi_{i,k\sigma }^{\text{el}*}(r,t)\nabla \phi_{i,k\sigma }^{\text{el}}(r,t)\right].$$ (25)

Using the relation between the expansion coefficients of the Floquet–Bloch functions at opposite **k** due to the time-reversal symmetry in Eq. (20), we obtain the time evolution of the electron current density

$$j(r,t)=-\sum_{\mu_{\text{odd}}\geq 1}j_{\mu_{\text{odd}}}(r)\;\text{cos}(\mu_{\text{odd}}\omega t)-\sum_{\mu_{\text{even}}\geq 2}j_{\mu_{\text{even}}}(r)\;\text{sin}(\mu_{\text{even}}\omega t),$$ (26)

where

$$j_{\mu_{\text{odd}}}(r)=2\sum_{\sigma }\int_{\text{HBZ}}\frac{d^{3}k}{V_{\text{uc}}}\text{Im}{\tilde{j}}_{k\sigma ,\mu_{\text{odd}}}+\frac{E_{\text{em}}(r_{0})}{2\omega }[\varrho_{\mu_{\text{odd}}-1}(r)+\varrho_{\mu_{\text{odd}}+1}(r)],$$ (27)

$$j_{\mu_{\text{even}}}(r)=2\sum_{\sigma }\int_{\text{HBZ}}\frac{d^{3}k}{V_{\text{uc}}}\text{Re}{\tilde{j}}_{k\sigma ,\mu_{\text{even}}}-\frac{E_{\text{em}}(r_{0})}{2\omega }[\varrho_{\mu_{\text{even}}-1}(r)+\varrho_{\mu_{\text{even}}+1}(r)],$$ (28)

with

$${\tilde{j}}_{k\sigma ,\mu }=\sum_{i,m,m\prime ,\mu \prime }c_{m^{\prime },k\sigma ,\mu^{\prime }+\mu }^{i*}c_{m,k\sigma ,\mu^{\prime }}^{i}(\varphi_{m\prime k\sigma }\nabla \varphi_{mk\sigma }^{*}-\varphi_{mk\sigma }^{*}\nabla \varphi_{m\prime k\sigma })$$ (29)

are real-valued amplitudes of the electron current density. Thus, the electron-current-density amplitudes oscillate with a phase shifted by π/2 with respect to the oscillations of the charge distributions of the same order. When the absolute value of the *μ*th-order charge oscillation is at a maximum, the *μ*th-order electron current density is zero and vice versa.

The expression of the electron current density in Eq. (26) is presented in velocity gauge. As shown in Ref. 54, the electron current density calculated in velocity gauge equals the electron current density in length gauge taking the sum of the interband and intraband contributions into account. The separate contributions of interband and intraband currents, however, must be corrected to be consistent in both gauges. Here, we discuss the total electron current density, not separate contributions.

Applying the continuity equation

$$\begin{array}{c} \text{div}j(r,t)=-\partial \rho (r,t)/\partial t\\ =\sum_{\mu_{\text{odd}}\geq 1}\mu_{\text{odd}}\omega \varrho_{\mu_{\text{odd}}}(r)\;\text{cos}(\mu_{\text{odd}}\omega t)+\sum_{\mu_{\text{even}}\geq 2}\mu_{\text{even}}\omega \varrho_{\mu_{\text{even}}}(r)\;\text{sin}(\mu_{\text{even}}\omega t), \end{array}$$ (30)

we obtain the following relation between the amplitudes of the electron current density and the amplitudes of the electron density:

$$\text{div}j_{\mu }(r)=-\mu \omega \varrho_{\mu }(r).$$ (31)

Let us now analyze the connection between the dipole moment of the optically induced charge distributions $\int d^{3}rr\varrho_{\mu }(r)$ and the electron-current-density amplitudes

$$\int d^{3}rr\varrho_{\mu }(r)=-\frac{1}{\mu \omega }\int d^{3}rr\text{div}j_{\mu }(r)$$ (32)

$$=-\frac{1}{\mu \omega }\oint r[j_{\mu }(r)\cdot dS]+\frac{1}{\mu \omega }\int d^{3}rj_{\mu }(r).$$ (33)

Equation (33) follows from vector–algebra relations for the dipole moment of a divergence. If the electron current density $j_{\mu }(r)$ on the boundary of a unit cell is zero, the first term in Eq. (33) disappears, and the *μ*th-order macroscopic polarization is proportional to the volume integral of the *μ*th-order electron-current-density amplitudes

$${\tilde{P}}^{\left(\mu \right)}(\mu \omega )\propto \int d^{3}rr\rho_{\mu }(r,t)\propto \int d^{3}rj_{\mu }(r).$$ (34)

### E. Optically induced charge distributions and electron current density in a crystal with inversion symmetry

In this subsection, we assume that the crystal exposed to periodic driving is invariant under inversion symmetry and, consequently, ${\hat{H}}_{\text{el}}(r)={\hat{H}}_{\text{el}}(-r)$, and derive the symmetry properties of electron density and electron-current-density amplitudes. The interaction of Hamiltonian ${\hat{H}}_{\text{int}}^{\text{cl}}$ is invariant under the transformations r→−r and t→t−T/2. Thus, it follows from the time-dependent Schrödinger equation in Eq. (15) that:^42^

$$i\frac{d\phi_{i,k\sigma }^{\text{el}}(-r,t-T/2)}{dt}=[{\hat{H}}_{\text{el}}+{\hat{H}}_{\text{int}}^{\text{cl}}(t)]\phi_{i,k\sigma }^{\text{el}}(-r,t-T/2).$$ (35)

Since $\phi_{i,k\sigma }^{\text{el}}(r+R,t)=e^{ik\cdot R}\phi_{ik\sigma }^{\text{el}}(r,t),\;\phi_{i,k\sigma }^{\text{el}}(-r,t-T/2)$ is a solution at −k and

$$\phi_{i,-k\sigma }^{\text{el}}(-r,t-T/2)=\phi_{i,k\sigma }^{\text{el}}(r,t).$$ (36)

The Bloch functions of crystals that are invariant under inversion symmetry obey the relation $\varphi_{mk\sigma }(r)=\varphi_{m-k\sigma }(-r)=\varphi_{mk-\sigma }^{*}(-r)$.^50^ Substitution of the relations between Bloch and Floquet–Bloch functions into the expansion of the Floquet–Bloch functions in Eq. (5) gives a relation between the expansion coefficients at opposite **k,**

$$c_{m,k↑,\mu }^{i}={\left(-1\right)}^{\mu }c_{m,-k↓,\mu }^{i}.$$ (37)

Comparing it with the relation between the coefficients due to time-reversal symmetry in Eq. (20), we find that the coefficients $c_{m,k\sigma ,\mu }^{i}$ are real.

Substitution of these properties into the expression for the optically induced charge distributions via Bloch functions in Eq. (7) leads to the following connection between complex amplitudes at opposite **r**:

$${\tilde{\rho }}_{\mu }(-r)={\tilde{\rho }}_{\mu }^{*}(r).$$ (38)

The property of these amplitudes that either their imaginary or real part is zero depending on the parity of *μ* determines how they behave under inversion symmetry. The same holds for the real-valued representation of the optically induced charge distributions in Eqs. (23) and (24) that all even-order optically induced charge distributions are invariant under inversion symmetry, whereas all odd-order optically induced charge distributions are opposite under inversion symmetry

$$\varrho_{\mu_{\text{even}}}(r)=\varrho_{\mu_{\text{even}}}(-r),$$ (39)

$$\varrho_{\mu_{\text{odd}}}(r)=-\varrho_{\mu_{\text{odd}}}(-r).$$ (40)

Analogously, using that $\varphi_{mk\sigma }(-r)=\varphi_{mk-\sigma }^{*}(r)$,^50^ $\nabla_{-r}=-\nabla_{r}$ and the coefficients $c_{m,k\sigma ,\mu }^{i}$ being real, we obtain the following symmetry properties of the current-density amplitudes:

$$j_{\mu_{\text{even}}}(r)=-j_{\mu_{\text{even}}}(-r),$$ (41)

$$j_{\mu_{\text{odd}}}(r)=j_{\mu_{\text{odd}}}(-r).$$ (42)

The volume integral of the even-order current density amplitudes is zero

$$\int d^{3}rj_{\mu_{\text{even}}}(r)=0,$$ (43)

in agreement with the selection rule that even-order harmonics from crystals invariant under inversion symmetry are forbidden.^55^

## III. MICROSCOPIC OPTICAL RESPONSE IN BANDGAP CRYSTALS MgO AND GaAs

### A. Computational details

We diagonalize the Floquet–Bloch Hamiltonian as described in Refs. 39 and 49. We calculate the one-body wave functions $\varphi_{mk\sigma }$ of the field-free Hamiltonian ${\hat{H}}_{\text{el}}$ with the density functional theory using the ABINIT software package^56–58^ in combination with Troullier–Martins pseudopotentials.^59^ The functions $\varphi_{mk\sigma }$ of valence bands and conduction bands are calculated on a dense grid of **k** points in half of the Brillouin zone. The numbers of blocks of the Floquet–Bloch matrix, **k** points, and bands are increased in the computations until convergence of the Fourier components of the optically induced charge distributions is reached. We apply the scissors approximation^60^ to correct a bandgap to its experimental value.

The conduction bands that are necessary to converge the optical response are actually the bands into which electrons are excited by the electromagnetic field with a nonvanishing probability. The number of conduction bands involved in the interaction with the optical field strongly depends on the intensity of the optical field and crystal properties. This number increases with the intensity of the optical field and is well above ten in the nonperturbative regime. There are several reasons for such a high required number of conduction bands. The first reason is that the higher the intensity of the optical field, the larger is the probability of an off-resonant transition into energetically high conduction bands. For example, a transition from a valence band into a conduction band with an energy difference detuned by 10 eV from the photon energy of the driving field can contribute to the first harmonic, if the intensity of the optical field is sufficiently high.

The next reason is that the higher the intensity of the optical field, the larger is the probability of a resonant multiphoton transition. As an example, let us consider the seven-photon absorption process induced by a field with a photon energy of ω=1.55 eV. A transition from a valence band to a conduction band with an energy difference of 7ω=10.85 eV is resonant and should have a dominating contribution to this process. Then, it is crucial to take into account the conduction bands lying at ≈11 eV above the outermost valence band for the calculation of the seventh-order optical response. The number of generated harmonics increases with increasing field intensity, and so should increase the number of conduction bands that are necessary to take into account for the calculation of a high harmonic spectrum. Multiphoton transitions also contribute to the optical response at lower orders. For example, seven-photon absorption combined with six-photon emission can contribute to the first-order response. Such contributions would become noticeable under conditions in which perturbation theory is likely to be no longer valid.

We used the same computational scheme for the calculation of an x-ray-optical wave-mixing signal in Ref. 49. We have shown that x-ray-optical wave-mixing signal is connected to the Fourier transform of optically induced charge distributions from the real space to the momentum space. That is why in that study, we focused on the calculation of optically induced charge distributions in the momentum space and did not analyze their real-space properties.

### B. Crystal with inversion symmetry, MgO

We first calculate the microscopic optical response of a crystal with inversion symmetry, MgO. We consider driving optical field with an intensity of $I_{\text{em}}=2\times 10^{12}$ W/cm^2^, a photon energy of 1.55 eV, and polarization axis ϵ=(0,0,1). The calculation is performed using a 24×24×24 Monkhorst–Pack grid, four valence and sixteen conduction bands, and 81 blocks of the Floquet Hamiltonian, which are necessary to reach convergence. The driving field and the computational parameters are the same as we used to calculate the subcycle-unresolved x-ray-optical wave-mixing signal from laser-dressed MgO in Ref. 49. There, we showed that an optical field of $2\times 10^{12}$ W/cm^2^ drives electron dynamics in MgO nonperturbatively. For the current computation, we additionally apply the scissors approximation^60^ to correct the bandgap from the calculated 5.6 eV to the experimental value of 7.8 eV.^61^

**FIG. 1.**
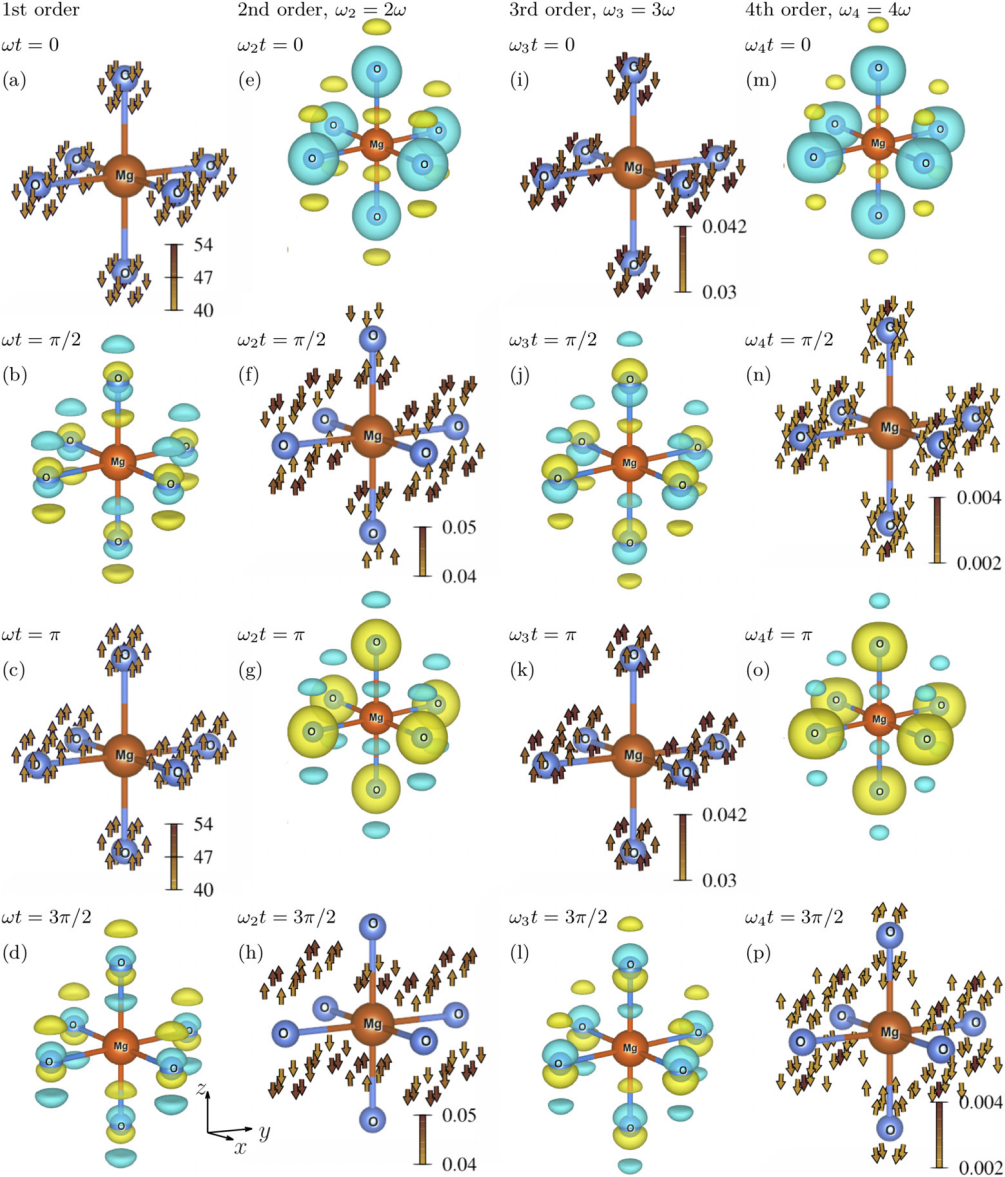
The *μ*th-order microscopic optical response of a MgO crystal at different phases of the driving electromagnetic field polarized along the *z* direction, where *μ* = 1, 2, 3, and 4. A cut of a unit cell centered around the Mg atom is shown. The first column shows the oscillations of the electron density and the electron current density with frequency *ω* at phases *ωt* equal to (a) 0; (b) π/2; (c) *π*; and (d) 3π/2. Second column corresponds to 2ω oscillations at phases *ωt* equal to (e) 0; (f) π/4; (g) π/2; and (h) 3π/4. Third column shows 3ω oscillations at phases *ωt* equal to (i) 0; (j) π/6 (k) π/3; and (l) 3π/6. Fourth column shows 4ω oscillations with at phases *ωt* equal to (m) 0; (n) π/8; (o) π/4; and (p) 3π/8. The yellow and blue colors represent negative and positive charges, respectively.

Figure 1 shows the calculated microscopic response of the MgO crystal depending on the phase of the driving electromagnetic field with the electric field evolving as $E_{\text{em}}\;\text{sin}(\omega t)$. A cut of a unit cell centered at the Mg atom is shown. The first column displays the first-order oscillations of the electronic state, i.e., the oscillations of the electron density and the electron current density with frequency *ω* in response to the driving electromagnetic field. As shown in Sec. II B, the first-order oscillations of the electronic state of the laser-driven crystal comprise the oscillations of the electron current density as $-j_{1}(r)\;\text{cos}(\omega t)$ and the oscillations of the electron density as $-\varrho_{1}\;\text{sin}(\omega t)$.

The first-order optically induced electronic state at ωt=0 is shown in Fig. 1(a). At this phase, the electric field of the optical field is zero, the magnitude of the microscopic first-order electron current is at a maximum, and the first-order electron density is zero. Thus, Fig. 1(a) shows only the first-order electron current density $-j_{1}(r)$. The *μ*th-order electron current amplitudes $j_{\mu }(r)$ are three-dimensional vector fields that are nonzero at most points within the unit cell. For the purpose of intuitive visual representation, the electron current densities in this and other figures are plotted on a sparse grid and only vectors with magnitudes $\left|j_{\mu }(r)\right|$ larger than a certain minimum threshold are shown. The magnitudes of $j_{\mu }(r)$ are color-coded, and their values are in atomic units. The minimum threshold for $\left|j_{\mu }(r)\right|$ in a given plot is the minimum value of the corresponding color box.

The first-order electron current density in Fig. 1(a) points along the driving-field polarization direction, and its magnitude $\left|j_{\mu }(r)\right|$ has pronounced peaks at the oxygen atoms. The electron current causes the charge to rearrange within the unit cell and, at ωt=π/2, the first-order electron density $-\varrho_{1}\;\text{sin}(\omega t)$ reaches the maximal magnitude.

Figure 1(b) shows the first-order electron density at ωt=π/2, when the electric field is at a maximum and the first-order electron current density is zero. The electron densities in this and other figures are represented in terms of an isosurface using VESTA software.^62^ The yellow and blue colors, respectively, represent negative and positive charges relative to the field-free electron density. As shown in Sec. II E, the optically induced charge distributions of a laser-driven crystal with inversion symmetry also have inversion symmetry. Thereby, odd-order optically induced charge distributions are antisymmetric with respect to a center of symmetry. Consistently, the values of the first-order electron density in Fig. 1(b) at positions **r** and −r relative to the Mg atom are opposite. The positive and negative charges in Fig. 1(b) alternate along the *z* axis parallel to the optical-field polarization. This charge alignment is consistent with the macroscopic first-order polarization of MgO being aligned with the electric field as expected.

At ωt=π, the magnitude of the first-order electron current density is again at a maximum, but opposite to that at ωt=0. The electron current now causes the charge to redistribute in the opposite direction. At ωt=3π/2, the first-order electron density is opposite to the first-order electron density at ωt=π/2. The macroscopic polarization is again aligned with the electric field.

The second column of Fig. 1 shows the second-order oscillations, i.e., the oscillations with frequency $\omega_{2}=2\omega$, of the electronic state in response to the driving field. The second-order oscillations of the electronic state of the laser-driven crystal are shown, comprising the oscillations of the electron current density as $-j_{2}(r)\;\text{sin}(2\omega t)$ and the oscillations of the electron density as $\varrho_{2}\;\text{cos}(2\omega t)$. The second-order macroscopic polarizability tensor of the MgO crystal is zero because of inversion symmetry. Surprisingly, we find that the second-order *microscopic* optical response is nonzero. In order to understand this phenomenon, let us look into the second-order electronic state at $\omega_{2}t=0$ shown in Fig. 1(e). At this phase, the second-order electron current density is zero and the magnitude of the second-order electron density is maximal.

As discussed in Sec. II E, an even-order optically induced charge distribution of a centrosymmetric crystal is also centrosymmetric. Consistently, the second-order electron density in Fig. 1(e) is centrosymmetric with respect to the Mg atom. Therefore, the charge distribution in Fig. 1(e) does not have a dipole moment and results in zero *macroscopic* polarization. Analogously, the general statement that an even-order optically induced charge distribution of a centrosymmetric crystal is also centrosymmetric is consistent with the selection rule that the even-order macroscopic polarization of such a crystal is zero.

Figure 1(f) shows the second-order electronic state at ωt=π/4, when the magnitude of the second-order electronic current density is at a maximum. Consistently with the discussion in Sec. II E, the microscopic second-order electron current density is antisymmetric with respect to the center of symmetry.

The third column of Fig. 1 shows the third-order oscillations of the electronic state of laser-driven MgO crystal, comprising the oscillations of the electron density as $-\varrho_{3}\;\text{sin}(3\omega t)$ and of the electron current density as $-j_{3}\;\text{cos}(3\omega t)$. Figure 1(i) shows the third-order electronic state at ωt=0, which is given by the third-order electron current density. Like the first-order electron current density in Fig. 1(a), $j_{3}(r)$ points predominantly in the direction of the driving-field polarization. Figure 1(j) shows the third-order electron density at ωt=π/6. Interestingly, it has a very similar structure to the first-order electron density in Fig. 1(b). The charge distribution in Fig. 1(j) also clearly indicates that the third-order macroscopic polarization points along the driving-field polarization direction.

The fourth column of Fig. 1 shows the fourth-order oscillations of the electronic state that comprise the oscillations of the electron density as $\varrho_{4}\;\text{cos}(4\omega t)$ and of the electron current density as $-j_{4}\;\text{sin}(4\omega t)$. The fourth-order electron density at ωt=0 shown in Fig. 1(m) has a very similar structure to the second-order electron density in Fig. 1(e). Since the fourth-order charge distribution is centrosymmetric, it leads to zero fourth-order macroscopic polarization.

It is possible to access optically induced charge distributions in an experiment. They can be probed by x-ray-optical wave mixing, in which x-ray diffraction is performed during the action of optical excitation of a crystal.^49,63,64^ We demonstrated that the x-ray-optical wave-mixing signal encodes the square of **G**th Fourier component of *μ*th-order optically induced charge distributions, $|\int d^{3}re^{iG\cdot r}\varrho_{\mu }(r){|}^{2}$.^49^ They determine the intensity of the *μ*th-order side peaks to a Bragg peak at the reflection **G** that is the signal separated by μω in energy and by μq in the momentum space from this Bragg peak, where q is the momentum of optical photon. In that study, we presented the calculation of an x-ray-optical wave-mixing signal from a laser-driven MgO as an example. We found several surprising phenomena that we could not explain. We can now understand them looking at the figures showing the microscopic optical response of MgO.

The first unexpected result was that the intensities of even-order side peaks that are proportional to $|\int d^{3}re^{iG\cdot r}\varrho_{\mu_{\text{even}}}(r){|}^{2}$ were nonzero, although even-order harmonics of MgO are zero. We now found that the microscopic even-order optical response of MgO is nonzero although it results in zero macroscopic optical response. In the x-ray-optical wave mixing experiment, x rays give access to the atomic scale and reveal the microscopic optical response.

The second surprising observation was that the intensities of the odd-order side peaks that are proportional to $|\int d^{3}re^{iG\cdot r}\varrho_{\mu_{\text{odd}}}(r){|}^{2}$ were zero at **G** perpendicular to the driving-field polarization ϵ, whereas the intensities of the even-order side peaks did not change considerably at G⊥ϵ. Comparing the odd-order and even-order amplitudes of the electron density, and the odd-order and even-order amplitudes of the electron current density in Fig. 1, this behavior becomes clear. The odd-order electron current densities are aligned along the driving-field polarization direction, such that $\int d^{3}re^{iG\cdot r}\varrho_{\mu }(r)=\int d^{3}re^{iG\cdot r}\text{div}j_{\mu }(r)=G\cdot \int d^{3}re^{iG\cdot r}j_{\mu }(r)$ is zero at G⊥ϵ. The even-order density amplitudes are close to a spherically symmetric distribution, and their Fourier components do not have such a pronounced angular dependence. That is why their Fourier components do not strongly depend on the angle between **G** and ϵ.

### C. Crystal without inversion symmetry: GaAs

We consider the microscopic optical response of a crystal without inversion symmetry, GaAs, which has a bandgap of 1.42 eV.^65^ We consider optical excitation by an optical field of 1 eV photon energy and an intensity of $4\times 10^{11}$ W/cm^2^. The factor $\sqrt{I_{\text{em}}}/\omega$ entering the off diagonal matrix elements of the Floquet Hamiltonian^39^ is the same as in the calculation of optical response of MgO. 28×28× 28 Monkhorst–Pack grid, 4 valence and 56 conduction bands, and 151 blocks of the Floquet–Bloch Hamiltonian are necessary to converge the results. According to the second-order susceptibility tensor of the space group F $\bar{4}$3m,^55^ the second-order macroscopic response of GaAs driven by a field polarized along the (1, 1, 1) direction is allowed, whereas, for a field polarized along the (1, 0, 0) direction, it is forbidden. In this subsection, we compare the microscopic optical response of GaAs to the excitation by driving fields polarized along the (1, 1, 1) direction and along the (1, 0, 0) direction.

In the presented calculations, we did not take spin–orbit coupling (SOC) into account. Using a smaller Monkhorst–Pack grid ( 4×4×4), we studied the role of SOC for the calculation of laser-dressed GaAs, since SOC plays a role for the electronic structure of GaAs.^66,67^ In the presence of SOC, bands cannot be assumed to be doubly occupied and we took 120 bands into account. We do observe that spin-up and spin-down components of optically induced charge distributions behave differently, but this effect is small. GaAs is invariant under time-reversal symmetry. Therefore, spin-averaged densities have either only nonzero real or only nonzero imaginary part (see Sec. II C). Comparing spin-averaged charge distributions obtained with and without SOC for driving fields polarized along the (1, 0, 0) direction, we saw that SOC does not play an essential for spin-averaged charge distributions.

**FIG. 2.**
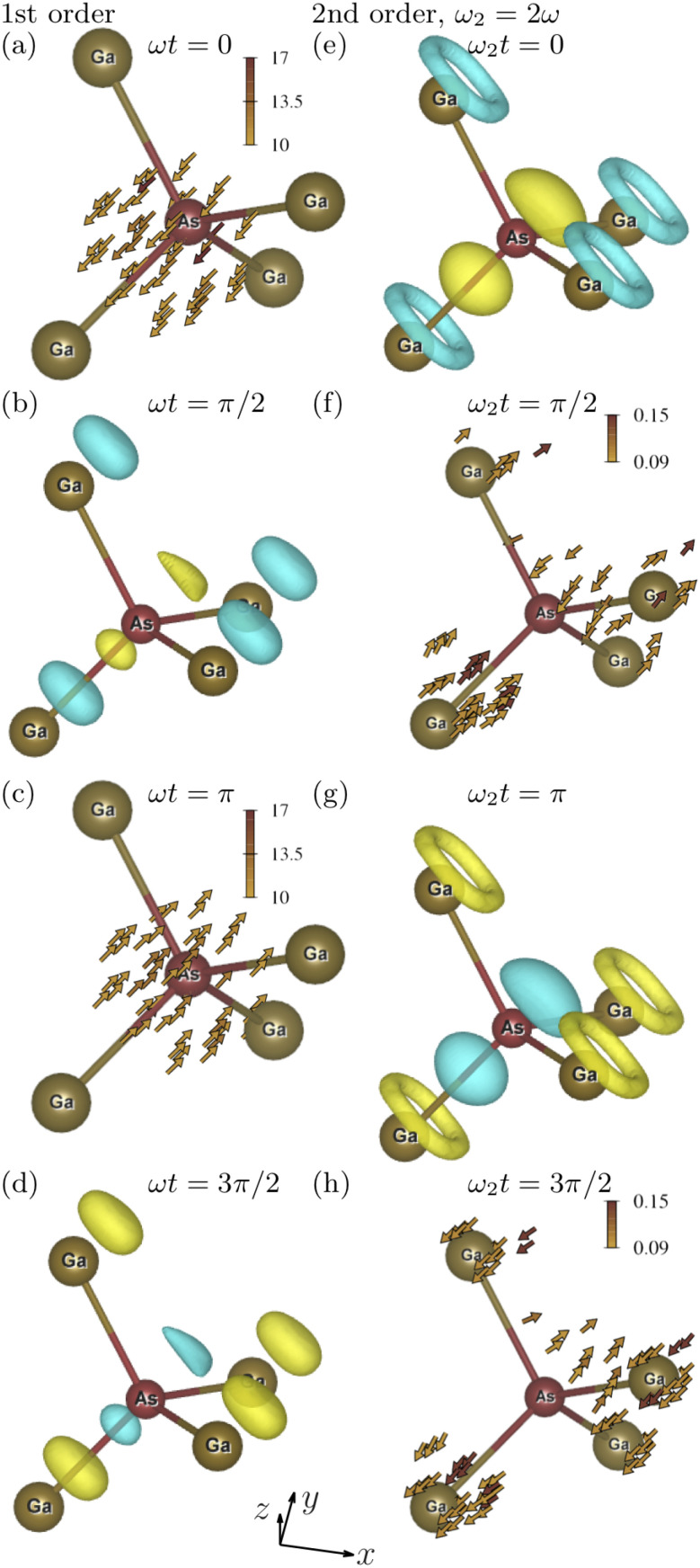
The first- and second-order microscopic optical response of a GaAs crystal at different phases of the driving electromagnetic field polarized along the (1, 1, 1) direction. A cut of a unit cell of GaAs centered at the As atom is shown. The first column shows the oscillations of the electron density and the electron current density with the frequency *ω* at phases *ωt* equal to (a) 0; (b) π/2; (c) *π*; and (d) 3π/2. Second column corresponds to 2ω-oscillations at phases *ωt* equal to (e) 0; (f) π/4; (g) π/2; and (h) 3π/4. The yellow and blue colors represent negative and positive charges, respectively.

The first and second columns of Fig. 2 show, respectively, the first- and second-order oscillations of the electronic state of GaAs driven by an optical field polarized along the (1, 1, 1) direction. The first-order oscillations of laser-driven GaAs comprise the oscillations of the electron current density as $-j_{1}(r)\;\text{cos}(\omega t)$ and the oscillations of the electron density as $-\varrho_{1}\;\text{sin}(\omega t)$.

Figure 2(a) shows the first-order electron current density at ωt=0. The vector field $-j_{1}(r)$ clearly points along the driving-field polarization direction in agreement with the selection rule that the macroscopic first-order polarization of GaAs is aligned with the electric field.^55^ Figure 2(b) shows the first-order electron density at ωt=0. It has a threefold rotational symmetry with respect to the driving-field polarization direction (1, 1, 1). The positive charge alternates with the negative charge along the (1, 1, 1) direction.

The second column of Fig. 2 shows the second-order oscillations of the electronic state that comprise the oscillations of the electron density as $\varrho_{2}\;\text{cos}(2\omega t)$ and of the electron current density as $-j_{2}\;\text{sin}(2\omega t)$. According to the second-order susceptibility tensor of GaAs,^55^ the second-order macroscopic polarization driven by an electric field polarized along the (1, 1, 1) direction is also aligned along (1, 1, 1). Figure 2(e) shows the second-order electron density at ωt=0. It also displays a threefold rotational symmetry with respect to the driving-field polarization direction (1, 1, 1). The positive charge alternates with the negative charge along the (1, 1, 1) direction in agreement with the macroscopic polarization aligned along (1, 1, 1).

The magnitude of the second-order electron current density reaches the maximum at ωt=π/4 and is shown in Fig. 2(f). It has a very complex structure that is difficult to characterize. We calculate the volume integral of $-j_{2}$ and find that it indeed points in the (1, 1, 1) direction in agreement with the selection rule for the second-order macroscopic polarization.

**FIG. 3.**
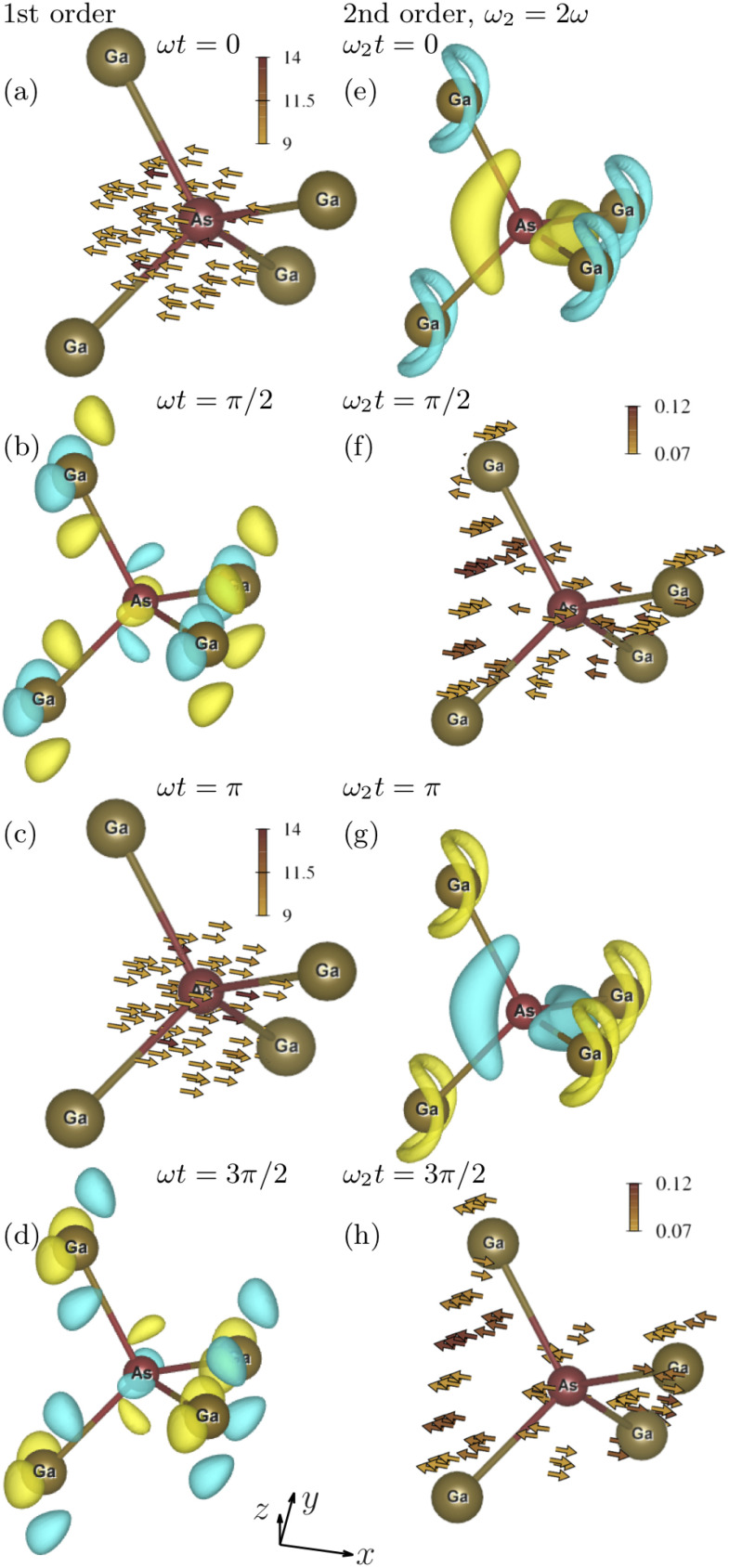
Same as for Fig. 2, except the driving field is polarized along the (1, 0, 0) direction.

The first and second columns of Fig. 3 show, respectively, the first- and second-order oscillations of the electronic state of GaAs driven by a field polarized along the (1, 0, 0) direction. Figure 3(a) shows the first-order electron current density at ωt=0, when its magnitude is at a maximum. The vector field clearly points along the driving-field polarization direction (1, 0, 0). This is in agreement with the alignment of the first-order macroscopic polarization of GaAs with the electric field.^55^

Figure 3(b) shows the first-order electron density at ωt=π/2. It has twofold rotational symmetry with respect to the direction of the driving-field polarization (1, 0, 0). It is not obvious how the charge distribution in Fig. 3(b) results in the first-order macroscopic polarization along (1, 0, 0), but one may notice that negative charges alter with positive charges in the *x* direction, when looking at charges around the bottom Ga atoms.

According to the second-order susceptibility tensor of GaAs,^55^ its second-order macroscopic polarization is zero for a driving field polarized along the *x* direction. We obtain that the second-order microscopic optical response is, in fact, nonzero. Figure 3(e) shows the second-order electron density at ωt=0. It also has twofold rotational symmetry about the *x* axis as $\varrho_{1}(r)$ in Fig. 3(b). Figure 3(f) shows the second-order electron current density at ωt=π/4. Despite its complex structure, we find in our calculations that its volume integral is indeed zero in agreement with the zero second-order macroscopic polarization. The magnitudes of the second-order electron current density in Figs. 3(f) and 3(h) are similar to the magnitudes of the second-order electron current density induced by the field polarized along the (1, 1, 1) direction in Figs. 2(f) and 2(h). However, in the first case, the volume integral over the second-order electron current density is negligible and, in the second case, it is considerable. This demonstrates that microscopic optical response resulting in vanishing macroscopic optical response and microscopic optical response resulting in considerable macroscopic optical response can have comparable magnitudes.

## IV. CONCLUSIONS

Macroscopic polarization and a HHG spectrum of a crystal are determined by the dipole moment of the electron density. On the atomic scale, optically induced charge distributions go far beyond the concept of a dipole and have a complex spatial structure. This structure has several interesting properties determined by the symmetry of the crystal. Time-reversal symmetry determines the phase of *μ*th-order oscillations of the optically induced charge distribution and the electron current density. We found that components of the electron density oscillate either in phase with the electric field or in phase with the vector potential depending on the parity of the oscillation order. Even-order charge distributions evolve as harmonics in phase with the vector potential of the optical field, whereas odd-order charge distributions evolve as harmonics in phase with the electric field. For the even- and odd-order amplitudes of the electron current density, this is opposite. Spatial inversion symmetry of the crystal leads to the spatial inversion symmetry of *μ*th-order optically induced charge distributions and electron-current-density amplitudes. Thereby, even-order electron-current-density amplitudes are antisymmetric with respect to the transformation r→−r, and odd-order electron-current-density amplitudes are symmetric. Even-order optically induced charge distributions are symmetric, and odd-order distributions are antisymmetric. As a result, odd-order optically induced charge distributions are aligned in such a way that macroscopic polarization is induced, and even-order distributions lead to a vanishing macroscopic polarization and, thus, vanishing even-order harmonics. Thus, even when macroscopic optical response is forbidden, charges still rearrange within the unit cell of the crystal. Our analysis within the Floquet–Bloch formalism and calculations in combination with the DFT provide the microscopic insight into optically induced charge distributions due to strong-field optical excitation and provide a deeper understanding of relevant properties of strong-field response such as HHG in crystals.

## Data Availability

The data that support the findings of this study are available from the corresponding author upon reasonable request.
